# CysDBase: a comprehensive database of cysteine post-translational modifications across protein sequence, structure, microenvironment, class, cellular localization, biological pathway, and taxonomy

**DOI:** 10.1093/database/baag021

**Published:** 2026-05-12

**Authors:** Devarakonda Himaja, Debashree Bandyopadhyay

**Affiliations:** Department of Biological Sciences, Birla Institute of Technology and Science, Pilani, Hyderabad Campus, Hyderabad 500078, India; Department of Biological Sciences, Birla Institute of Technology and Science, Pilani, Hyderabad Campus, Hyderabad 500078, India

## Abstract

The reactive thiol group of cysteine (Cys) acts as a nucleophile and undergoes many cysteine post-translational modifications (Cys-PTMs). Cys-PTMs, called protein redox switch, contribute to various cellular and physiological processes, including reactive oxygen species (ROS)-induced signalling, ROS mitigation, and scavenging. Consolidation of Cys-PTMs into a database would facilitate the mechanistic elucidation of biological processes and therapeutic applications. The existing databases store information on cysteine motifs, oxidation states, a few of the Cys-PTMs, etc., specific to species or kingdoms, and lack general applicability. There was no mention of the impact of the protein microenvironments and cellular localizations on the Cys-PTMs. The current study reports a database containing 7 Cys-PTMs (disulphide, *S*-nitrosylation, *S*-palmitoylation, *S*-glutathionylation, *S*-sulphenylation, metal-binding, and thioether), 11 features, 33 06 395 UniProt IDs, and 1 14 56 639 cysteine residues, across the taxonomy, encompassing cellular organelles, enzyme classes, sequence motifs, protein structures, and microenvironments. The maximum number of cysteine residues is reported here compared to 16 contemporary cysteine databases. Twenty-one types of metal-binding cysteines and thioether modifications are reported for the first time. Enzyme classes, cellular localization, taxonomic preferences, and microenvironment around Cys-PTMs were systematically analysed and curated, indicating the pathogenic involvement of those Cys-PTMs. The database has a web access (https://cysdbase.bits-hyderabad.ac.in/) and a programmatic access via GitHub link (https://github.com/devhimd19/CysDBase). The query inputs to the repositories are UniProt ID, biological pathway, location, or genus name. Query outputs are 11 biological features, namely, protein name, Cys-PTMs, cysteine residue number, cysteine sequence motif, cell organelle, biological pathway, protein microenvironment (buried fraction and relative hydrophobicity [rHpy]), EC number and enzyme class, secondary structure, organism, and PubMed ID.

## Introduction

Cysteine (Cys) has a reactive thiol group that acts as a nucleophile and participates in various biological functions [1]. The cysteine thiol group undergoes a wide range of oxidations, leading to two broad categories of chemical reactions: a) post-translational modification (*in vivo*) and b) external agent-induced oxidation (*in vitro*). Cysteine post-translational modifications (Cys-PTMs) include disulphide, *S*-glutathionylation, *S*-nitrosylation, *S*-sulphenylation, *S*-palmitoylation, thioether, metal-binding, etc. Disulphide modification is formed between two free thiol groups from the same protein chain (*intra-disulphide*) or two different protein chains (*inter-disulphide*). It contributes towards protein folding and maturation of the extracellular domains of both membrane and secreted proteins [2]. *S*-glutathionylation is often induced by oxidative stress and acts as a biological switch to trigger oxidative signalling [3]. *S*-nitrosylation, induced by nitric oxide (NO), triggers epigenetic processes by transcriptional regulation of histone acetylation or deacetylation [4,5]. *S*-lipidation is another type of Cys-PTM where an acyl group (*long-chain fatty acid*) is added to the cysteine thiol group. *S*-palmitoylation is the most common type of *S*-lipidation, involved in protein–membrane association and relocation of modified proteins within the cell membrane [6]. *S*-sulphenylation is induced by reactive oxygen species (ROS) molecules, like H_2_O_2_, and acts as a key sensor to oxidative stress, non-enzymatic oxidative folding regulation, cell signalling, and signal transduction [7,8]. Thioether modification, forming a carbon–sulphur bond between a cysteine thiol group and another protein or ligand, is observed as a metabolite in many biological processes and prevents proteolytic degradation [9]. Cysteine metal-binding is primarily involved in proteins from the electron transport chain, metalloproteins, heavy metal scavenging, etc. [10]. Thus, Cys-PTMs are crucial in various biological functions. Annotation of the Cys-PTMs is crucial for mechanistic elucidations. However, experimental annotation of Cys-PTMs is time-consuming and expensive. Hence, many Cys-PTM prediction algorithms were developed based on protein sequences and structures. Some of these can predict multiple Cys-PTMs [11], and others can predict single Cys-PTM [12,13]. Cys-PTMs were consolidated into various databases [12–18]. Although some prediction databases can predict multiple Cys-PTMs, namely, CysModDB and iCysMod, those were limited to eukaryotes only. The published databases were mainly specific to functional properties, such as cysteine motifs, cysteine oxidations, Cys.Sqlite [19], or specific to a particular species, such as *Homo sapiens*, CysDB [20]. Cys-PTMs in the entire taxonomic world were unexplored. None of the existing databases reported the thioether, *S*-sulphenylation, and a variety of metal-binding modifications; nor those databases characterized cysteine protein microenvironments.

**Figure 1:**
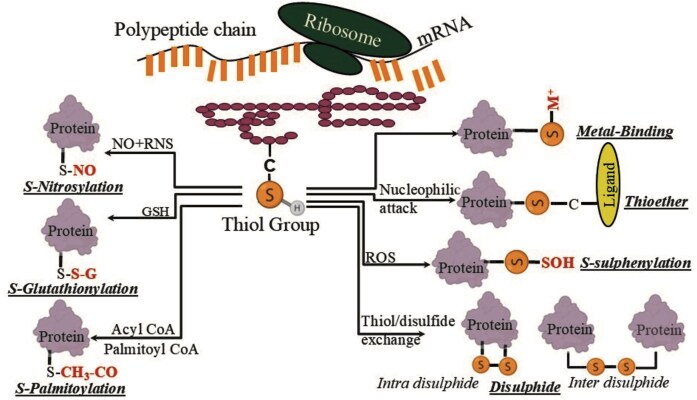
Different post-translational modifications of the cysteine residue in this study.

Here, we report a consolidated database of Cys-PTMs that includes seven cysteine post-translational modifications, namely disulphide, *S*-nitrosylation, *S*-palmitoylation, *S*-glutathionylation, *S*-sulphenylation, metal-binding, and thioether (Fig. 1). This database, for the first time, reports Cys-PTMs across the taxonomic world, analyses cysteine protein microenvironments, reports localization of the Cys-PTMs in cell organelles, and presents novel annotation for thioether modifications, not reported earlier. The following are the queries for the database: UniProt ID, biological pathway, location, or genus name. The query outputs are protein name, Cys-PTM, cysteine residue number, short sequence stretch with central cysteine of interest, cell organelle, biological pathway, protein microenvironment (buried fraction and relative hydrophobicity [rHpy]), EC number and enzyme class, organism, and PubMed ID [21].

## Materials and Methods

### Cys-PTM data curation

There were 11 Cys-PTMs reported in the UniProt database; however, sufficient information was available only for 7 Cys-PTMs, namely disulphide, *S*-glutathionylation, *S*-palmitoylation, *S*-nitrosylation, thioether, metal-binding, and *S*-sulphenylation. The information on these seven Cys-PTMs was curated mainly from the UniProt database [22]. A list of keywords was used (https://ftp.uniprot.org/pub/databases/uniprot/current_release/knowledgebase/complete/docs/keywlist.txt) from UniProt to extract the information of seven Cys-PTMs (Table 1; Table S1). The keyword list for thioether was presented separately as the keyword list was long (Table S1). Note that all the keywords were case-sensitive. As very little information was available on *S*-glutathionylation from UniProt, additional information was curated from the PGluS database [21,23]. The information extracted from the Uniprot database was of five types based on the evidence for the existence of a protein, namely i) experimental evidence at protein level, ii) experimental evidence at transcript level, iii) protein inferred from homology, iv) protein predicted, and v) protein uncertain (https://www.uniprot.org/help/protein_existence). The first two types are experimental in nature, thus, fewer in number, given the complexity of the experiments. Most of the Cys-PTMs reported in UniProt database were inferred from the homologous proteins. To note, one protein sequence represented by an UniProt ID multiple types of Cys-PTMs percentage of different types of existence of protein evidence was shown (Table S2).

**Table 1:** The keywords used to extract Cys-PTM information from the UniProt database.^a^

Cys-PTM	Keywords
Disulphide	‘Disulfide bond’
*S*-glutathionylation	‘*S*-glutathionyl cysteine’
*S*-sulphenylation	‘Cysteine sulfenic acid (-SOH)’
*S*-nitrosylation	‘S-nitrosocysteine’
Metal-binding: iron–sulphur clusters	‘2Fe-2S’, ‘3Fe-4S’, ‘4Fe-4S’, ‘Metallothioneins’, ‘8Fe-7S’, ‘7Fe-Mo-9S-C-homocitryl cluster’, ‘7Fe-V-9S-C-homocitryl cluster’, and ‘8Fe-9S-C-homocitryl cluster’
Metal-binding: nickel–iron sulphur clusters	‘Ni-Fe-S’, ‘Ni-4Fe-4S’, and ‘Ni-4Fe-5S’
Metal-binding: metal ions	‘Cd’, ‘Co’, ‘Cu’, ‘Hg’, ‘Na’, ‘K’, ‘Zn’, ‘Ca’, ‘Fe’, ‘Mg’, ‘Mn’, ‘Mo’, ‘W’, and ‘Ni’
*S*-lipidation	‘S-farnesyl cysteine’, ‘S-palmitoyl cysteine’, ‘S-geranylgeranyl cysteine’, ‘S-(15-deoxy-delta12,14-prostaglandin J2-9-yl) cysteine’, ‘N-palmitoyl cysteine’, and ‘S-diacylglycerol cysteine’.

a The keyword phrases are shown in quotes.

### Database construction

**Figure 2:**
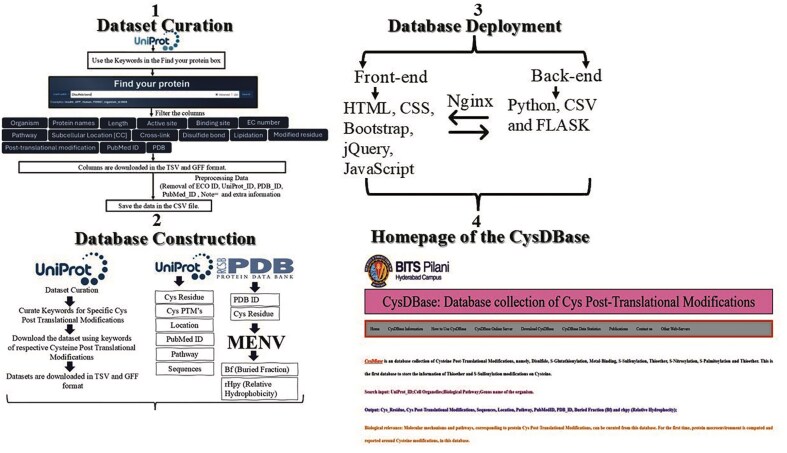
Schematic representation of dataset curation, database construction, deployment, and homepage of CysDBase.

The information retrieved from the iterative search of the UniProt database (saved either in a tab-separated value [TSV] or general feature format [GFF]) was customized; the customized columns were UniProt_ID, chemical cross linking, disulphide bond, lipidation, modified residue, post-translational modification, organism, length, protein names, pathway, subcellular location, EC number and class, PubMed ID, sequence, and PDB_ID. Further cleaning and processing of the dataset were done by removing duplicate information on ECO ID, UniProt ID, PDB_ID, PubMed ID, Note=’, etc. using an in-house AWK programmatic filtering. Based on the sequence and structure analyses, additional features were added to the customized table, namely a) a cysteine sequence motif and b) the predicted secondary structures based on the sequence motifs. The final table was saved as a comma-separated value file (CSV) (Fig. 2).

### CysDBase deployment

The home page of the CysDBase database was deployed using the FLASK web framework (3.0.3) written in Python (3.8.10). Reverse-based proxy server NGINX (1.18.0) and Gunicorn (20.0.4) were used. Web Server Gateway Interface (WSGI) communicates between the web server and the Python web application (Fig. 2).

*Description of the features in the database*:

The database returned a total of 11 features as a response to the user-defined queries, namely i) taxonomic kingdom, ii) protein name, iii) protein length, iv) EC number and EC class, v) biochemical pathway, vi) subcellular location, vii) post-translational modifications, viii) PubMed ID, ix) window size sequence, x) PDB ID, and xi) protein microenvironment. Each feature is described below.

- Taxonomic kingdom classification: The organism name available in the above-mentioned CSV file (Fig. 2) was matched with the UNIPROT taxonomic classification (https://ftp.uniprot.org/pub/databases/uniprot/current_release/knowledgebase/complete/docs/speclist.txt)—Archaebacteria, bacteria (prokaryota and eubacteria), eukaryota (Eukarya), viruses, and phages (Viridae). So far, no databases have included species across the taxonomic world.
- Protein names: Directly (without derivation or analysis) presented in the CSV file.
- Length of the protein: The protein length was represented by the number of amino acids (AA) and directly presented in the CSV file.
- EC number and EC class: The EC number for a particular UniProt ID follows a certain format, e.g. UniProt ID: A1L3X0 has an EC number: 2.3.1.199, where the first number signifies the class, and the following numbers represent the subsequent subclasses of the enzymes. The class of the enzyme is taken to denote the EC class. There are seven classes of enzymes, all reported in this study (Table 2).
- Biochemical pathway:

**Table 2:** Enzyme classes and their descriptions.

Enzyme class	Description
Oxidoreductases	Involved in oxidation and reduction processes
Transferases	Transfer of a group from one substrate to another
Ligases	Formation of new bonds and joining of two molecules
Lyases	Non-hydrolytic removal of functional groups from the substrate
Hydrolases	Hydrolytic reactions
Isomerases	Isomerization reactions
Translocases	Movement of ions or molecules across membranes or their separation within membranes

Three categories of biochemical pathways, namely pathway, subpathway, and enzymatic reaction (step), as defined by UniProt (https://ftp.uniprot.org/pub/databases/uniprot/current_release/knowledgebase/complete/docs/pathlist.txt), were considered in this study (Table 3). Here, we assign the biochemical pathway name to each Cys-PTM. A cysteine may undergo post-translational modifications in multiple biochemical pathways.

- Subcellular location:

**Table 3:** Pathway types involved in the CysDBase curated from the UniProt database.

Types of pathway	Description
Subpathway	Describes that the protein is involved in a part of a pathway
Pathway	Describes that the protein is mainly involved in the main pathway
Enzymatic reaction (step)	Describes the protein involved in an enzymatic reaction or step of a particular pathway

A protein may migrate from one subcellular location (i.e. cell organelle) to another, or the protein can be expressed in a specific subcellular location, such as the cell membrane, Golgi apparatus, endoplasmic reticulum, and cytoplasm. The same cysteine may have multiple modifications in different cell organelles.

- Post-translational modification:

Cys-PTMs were curated from the UniProt database based on experimental conditions, conformational changes, etc. A single cysteine may undergo multiple modifications, as illustrated (Table 4).

**Table 4:** An example of cysteine multiple modifications on one UniProt ID.

UniProt_ID	Disulphide (C_1_–C_2_)	*S*-glutathionylation	*S*-nitrosylation	*S*-sulphenylation
P0ACQ4	C_180_–C_259_; C_199_–C_208_	C_199_	C_199_	C_199_

The multiple modifications for a single cysteine were also reported in the literature (Fig. S1). In the human gamma F_1_–F_0_ ATP synthase protein, Cys_251_ may undergo four different types of cysteine modifications under different conditions: a) disulphide bond formation with Cys_201_, under oxidative stress, b) *S*-glutathionylation, c) *S*-sulphenylation in the presence of H_2_S, and d) *S*-nitrosylation in the presence of NO [24,25].

- PubMed ID:

The PubMed IDs for all the entries were curated from UniProt and consolidated in this study.

- Window size sequence:

The protein FASTA sequences were downloaded from the UniProt database. A short sequence (window size) was extracted on both sides of the test cysteine to understand the sequence effect on a certain cysteine residue. The window size was benchmarked elsewhere, with the optimal value of seven [11]. A window size of *n* = 7 constitutes 15 (2*n* + 1) amino acids with a central cysteine (Fig. S2).

- PDB-ID:

The PDB_ID information has been curated from the PDB database [26], hyperlinked to the individual UniProt IDs. To note, each UniProt ID may correspond to multiple PDB files, differing in terms of protein length, experimental conditions, ligand binding, amino acid substitutions, etc.

- Protein microenvironment (MENV) calculations: Protein microenvironment (MENV) was computed for each cysteine in this study based on the protein crystal structures obtained from the above-mentioned PDB IDs. All the PDB IDs corresponding to individual UniProt ID were exploited to compute MENV around each cysteine residue that ensures sufficient sampling of the cysteine protein microenvironments. MENV was described in terms of two features, namely buried fraction (BF) and relative hydrophobicity (rHpy) values [27]. The buried fraction (BF) is the normalized surface area of the cysteine thiol group buried inside the protein; the value ranges from 0.0 (completely exposed to the solvent) to 1.0 (completely buried). Relative hydrophobicity (rHpy) is a relative measure of hydrophobicity (or hydrophilicity) around any amino acid or its side chain contributed by the protein structure (Hpy*A*) and the surrounding solvent molecules (Hpy*S*). The hydrophobic contribution was computed as the weighted (weighted by buried fraction) summation of Rekker’s fragmental constants (Fb) from the protein structure and the solvent contribution within the first contact shell of an amino acid (set of amino acids b) or its functional group (THpy) (Equations 1–3).
  (1) \begin{eqnarray*}
\textit{HpyA}\ = \ \mathop \sum \nolimits_a^{NA} \ \mathop \sum \nolimits _{Bb}^{NB} {\mathrm{Fb}}
\end{eqnarray*}
   
  (2) \begin{eqnarray*}
\textit{THpy} = BF*\textit{HpyA} + \left( {1 - BF} \right)*\textit{HpyS}
\end{eqnarray*}
   
  (3) \begin{eqnarray*}
\textit{rHpy} = \ \frac{{\textit{THpy}}}{{\textit{Hpys}}}
\end{eqnarray*}

Protein microenvironment space (constituted by BF and rHpy) around the cysteine thiol group was clustered using K-means clustering implemented using a Python script (3.12) and enabled with Scikit-learn (1.6.1) and matplotlib (3.10.3) [28–30]. The K-means algorithm clustered similar data points into three clusters (predefined number of groups) by iteratively assigning data points to the closest cluster and optimizing the cluster’s centroid position. All these features were consolidated (in a CSV file) to create the database, CysDBase (Fig. 2).

## Results

Statistics of the CysDBASE database.

### Cysteine post-translational modifications (PTMs) involved in biochemical pathways

CysDBASE contains 3 306 395 UniProt IDs in a CSV file. Multiple cysteine residues were curated from each Uniprot ID, resulting in 11.4 million (*n* = 114 56 639) cysteine residue entries. Each cysteine residue may undergo single or multiple post-translational modifications (PTMs), a total of seven reported here (Table 5).

**Table 5:** Statistics of cysteine post-translational modifications.

Cysteine post-translational modification	Number of cysteines
*S*-nitrosylation	911
*S*-glutathionylation	2418
*S*-palmitoylation	8564
Thioether	12 626
*S*-sulphenylation	143 802
Disulphide	1 717 922
Metal-binding	9 572 720
Total	11 456 639

**Figure 3:**
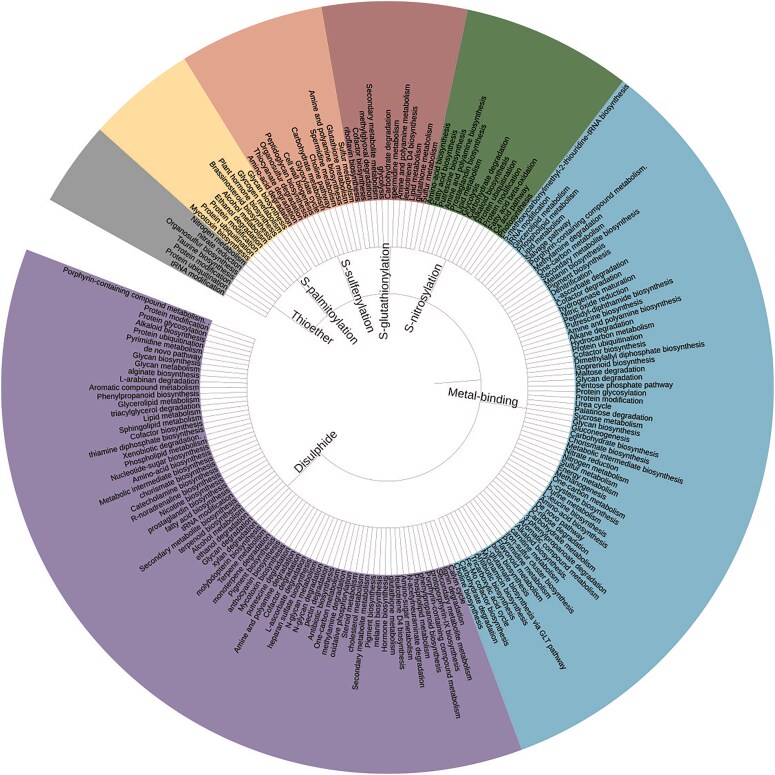
Simple tree representation of biochemical pathways involving seven Cys-PTMs (inwards). The pathway names appearing in >50 cysteine entries are shown for clarity (outwards). The figure was generated using Interactive Tree of Life (iTOL) v7.

The metal-binding PTMs were highest in number, 9.5 million (*n* = 95 72 720), and distributed across 21 different metal ions. The thioether PTM was reported for the first time. Seven Cys-PTMs involved in various biochemical pathways were reported here as a simple tree (Fig. 3).

**Figure 4:**
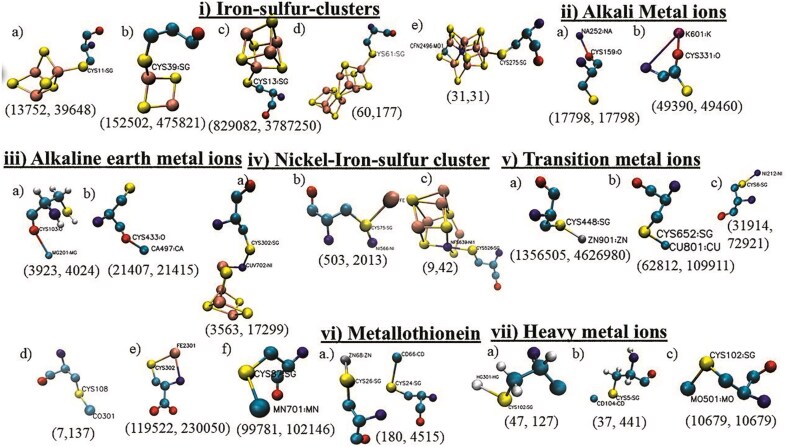
Seven different types of cysteine metal-binding: i) iron–sulphur cluster, ii) alkali metal ions, iii) alkaline earth metals, iv) nickel–iron sulphur clusters, v) transition metal ions, vi) a) metallothionein, and vii) heavy metal ions. Each type has several subtypes:—**i)** a) 3Fe–4S, b) 2Fe–2S, c) 4Fe–4S, d) 8Fe–7S, and e) 7Fe–Mo–9S–C-homocitryl cluster, **ii)** a) Na^+^ and b) K^+^, **iii)** a) Mg^2+^ and b) Ca^2+^, **iv)** a) Ni–4Fe–4S, b) Ni–Fe–S, and c) Ni–4Fe–5S, **v)** a) Zn^2+^, b) Cu^2+^, c) Ni^2+^, d) Co^2+^, e) Fe^2+^, and f) Mn^2+^, **vi)** metallothionein, **vii)** a) Hg^2+^, b) Cd^2+^, and c) Mo^2+^. The number of UniProt IDs and the number of cysteines are shown within parentheses below each subtype.

Metal-binding:

Twenty-one different types of metal-binding cysteines were categorized into seven different groups: i) iron–sulphur cluster, ii) alkali metal ions, iii) alkaline earth metal ions, iv) nickel–iron–sulphur clusters, v) transition metal ions, vi) metallothionein, and vii) heavy metal ions (Fig. 4). The number of UniProt IDs and the number of cysteines involved in each subtype are reported. Cysteine residues coordinated to alkali and alkaline earth metal ions were reported for the first time. Transition metal ions and metal clusters coordinated to cysteine exhibited specific coordination geometries (Fig. 4i, iv, and v); whereas cysteine-alkali metal and alkaline earth metals showed non-specific interactions (Fig. 4ii and iii). Metallothionein is a special type of cysteine metal-binding where a metal–thiolate cluster is formed (Fig. 4vi). Cysteine metal-binding reported here was mainly involved in the co-factor biosynthesis (*n* = 772 784) and *de novo* or pyrimidine biosynthesis (*n* = 223 653). Cysteine metal-binding analyses were not elaborated elsewhere.

The most common cysteine-binding metal ion, Zn^2+^, was mainly observed in zinc finger motifs and alcohol dehydrogenase enzymes. The optimum pairing of Zn^2+^ and cysteine residue is due to the affinity of the soft base (thiolate ion of cysteine side chain) towards the soft acid (Zn^2+^) [31]. Cysteine affinity towards Zn^2+^, Cu^2+^, and Cd^2+^ ions was experimentally demonstrated using affinity chromatography [32]. Cysteine effectively scavenges many heavy metal ions, including Cd^2+^ and Hg^2+^ (soft acid). Cysteine–heavy metal ion (Cd^2+^, Hg^2+^, Ni^2+^, Zn^2+^, Fe^2+^, Mn^2+^, Cu^2+^, Mo^2+^, and Co^2+^) affinity was also reported earlier [33]. Iron, being a transition metal ion, has a higher affinity towards the cysteine thiolate group, forming many Fe–S clusters, often including nickel and molybdenum ions (Fig. 4). 4Fe–4S clusters were mainly observed in the Krebs cycle and electron transport chains, 8Fe–7S cluster in nitrogen fixation pathways, and Ni–Fe–S clusters in methanogenesis and one-carbon metabolism [34]. Unlike the transition metal ions, cysteine has a weaker affinity towards alkali and alkaline earth metal ions (hard acids). Details of the biochemical pathways involving cysteine metal-binding were shown (Fig. 3).

Disulphide:

Disulphide modifications link two thiol groups from two different cysteines present either on the same chain (*intra-disulphide bond*) or from two different chains (*inter-disulphide bond*), stabilize protein quaternary structure, and assist protein folding, often mediated by protein disulphide isomerase (PDI). Most of the disulphide modifications belong to hemagglutinin (HA) (*n* = 96 588) and neuraminidase (NA) (*n* = 72 152), in this database. Details of the biochemical pathways involving disulphide modifications were shown (Fig. 3). Disulphide modification is more or less ubiquitous across the pathways—predominant in biosynthesis (*n* = 36 777), metabolic (*n* = 41 381), degradation (*n* = 7054), protein modification (*n* = 19 025), and protein ubiquitination (*n* = 69).

*S*-nitrosylation:

*S*-nitrosylation is a Cys-PTM where an NO molecule is covalently linked to the cysteine thiol group, forming *S*-nitrosothiol (SNO). This PTM is an important component of reactive oxygen Species (ROS) and reactive nitrogen species (RNS) homeostasis in plants [35]. Details of the biochemical pathways curated in this database are shown (Fig. 3).

Lipid modification:

Lipid modification is a collection of a large number of modifications in proteins involving the cysteine thiol group and different lipid groups. Different lipid modifications were *S*-palmitoyl cysteine (*S*-palmitoylation), *N*-palmitoyl cysteine (*N*-palmitoylation), *S*-diacylglycerol cysteine, *S*-farnesyl cysteine (*S*-farnesylation), *S*-(15-deoxy-delta12,14-prostaglandin J2-9-yl) cysteine, and *S*-geranylgeranyl cysteine (*S*-geranylation) [36]. This PTM is an important component in membrane transportation [37]. Details of the biochemical pathways involving cysteine lipid modifications were shown (Fig. 3). Glycan biosynthesis (*n* = 3978) and glycogen metabolism (*n* = 3978) within the cell membrane are the predominant pathways that involve lipid modifications. Note that the number of cysteines in glycan biosynthesis and glycogen metabolism was identical, as those were from the same protein, phosphorylase b kinase regulatory subunit. Despite the availability of lipid modification prediction tools, no related database is available.

*S*-glutathionylation:

*S*-glutathionylation in proteins involves the cysteine thiol group linked to the glutathione (GSH) molecule; the modification is important in energy metabolism and glycolysis [12]. In the current curation, the number of *S*-glutathionylation is less. Moreover, biological pathways for many of the proteins were not reported in the UniProt database. The most abundant *S*-glutathionylation proteins reported here were glutathione-*S*-transferases. *S*-glutathionylation was mainly involved in glutathione and sulphur metabolism. Details of the biochemical pathways involving *S*-glutathionylation were shown (Fig. 3). *S*-glutathionylated proteins, along with *S*-nitrosylation, inhibit the irreversible oxidation of cysteine under oxidative stress, as exemplified in *Arabidopsis thaliana*, in the presence of abscisic acid and under water-deficient conditions [38].

Thioether:

In the thioether modification, cysteine is bonded to a carbon atom (Fig. 1) from a large number of ligands (Table 2). This is involved in several metabolic processes, namely organosulphur synthesis, protein modification, protein ubiquitination, etc. [39]. Details of the biochemical pathways curated in this database are shown (Fig. 3). Thioether modifications in nitrogen metabolism (*n* = 2) and tRNA modification (n = 6) are predominantly reported in this database.

*S*-sulphenylation:

In *S*-sulphenylation, the cysteine thiol group is oxidized to sulfenic acid (–SOH). A cysteine thiol may undergo a large number of oxidation states (ranging from −2 to 6); however, *S*-sulphenylation is the predominant form under oxidative stress [40]. *S*-glutathionylation often prevents further oxidation of the cysteine thiol group. Details of the biochemical pathways curated in this database are shown (Fig. 3).

**Figure 5:**
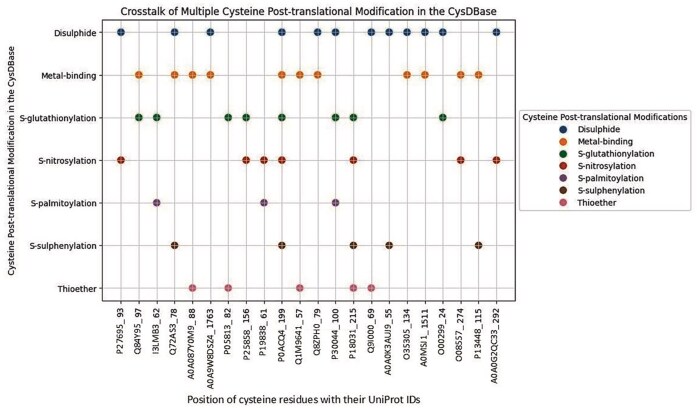
Cysteine cross-talk reported in the CysDBase. The X-axis represents UniProt IDs were followed by residue numbers (UniProt_Residue No.) and the Y-axis represents Cys-PTMs.

Cysteines with multiple PTMs (cross-talk) and their involvement in biochemical pathways:

Multiple Cys-PTMs (cross-talk) were observed for 21 cysteine residues in the current database. Most of the cross-talks were limited to two modifications (Fig. 5; Table S3). All these reported cross-talks were obtained from different experiments at the protein level, namely Edman sequencing, mass spectrometry, X-ray crystallography, Nuclear Magnetic Resonance (NMR) spectroscopy, and antibody detection, as reported in the UniProt database. Note that only five PTMs were annotated experimentally. Cysteine cross-talks were observed across different biochemical pathways, such as the electron transport chain and glycolysis. Many of these cross-talks happened due to oxidative stress or a downstream process, mainly in the cytoplasm, mitochondria, nucleus, endoplasmic reticulum, Golgi apparatus, cell membrane, and peroxisome [38]. For example, disulphide bonds in lens crystallin protein lead to aggregation and cataract formation [41]. On the other hand, *S*-glutathionylation or thioether formation prevents cataracts by inhibiting disuphfide formation in β-crystallin A3 (Table S3). Another example is GAPC1, where Cys_156_ is present at the catalytic site and is susceptible to oxidation (Fig. 5). Either glutathionylation or nitrosylation of Cys_156_ prevents further oxidation of the thiol [42]. Similarly, all the cysteines involved in cross-talk have either structural or functional consequences. These cysteine cross-talks often evolved in response to disease onsets, like cancer pathways involving Nuclear Factor kappa-light-chain-enhancer of activated B cells (NF-κB) [43]. For example, in osteoporosis and rheumatoid arthritis, there is an imbalance in the receptor activator of the NF-κB (RANK)–RANK ligand (RANKL) interaction. The RANK protein has four cysteine-rich domains where the backbone of Cys_134_ was coordinated to the sodium ion, providing structural stability [44]. The side chain of the same residue was involved in disulphide bond formation with Cys_152_ (UniProt ID: O35305); impairment of either led to disease. Details of the cross-talks were reported (Table S3). The graphical representation of the cross-talk in individual proteins was shown (Fig. 5). Note that this study is limited to seven Cys-PTMs; however, cross-talks might not be limited to these PTMs. An earlier report has presented cross-talk for oxidation, *S*-nitrosylation, *S*-glutathionylation, *S*-sulphenylation, *S*-sulfhydration, disulphide, *S*-palmitoylation, and *S*-sulfinylation [15]. Here, for the first time, we reported cross-talk between metal-binding and thioether.

### Cysteine post-translational modifications observed across the taxonomic kingdoms

**Figure 6:**
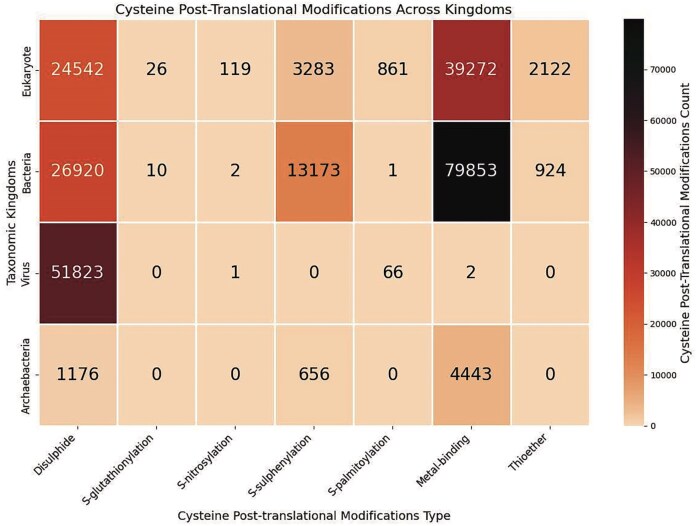
Heatmap of the cysteine post-translational modifications across various taxonomic kingdoms, in this study. The figure was generated using Python (version 3.12.3), module seaborn (version 0.13.2), and matplotlib (version 3.10.1).

Seven Cys-PTMs were classified across the taxonomic world (Fig. 6). All these Cys-PTMs were present in eukaryotes, mostly in plants, such as *A. thaliana*.

Bacterial species exhibited the maximum number of metal-binding cysteines. Most of the bacterial species involve metallothionein modification (*n* = 15 861). Metallothionein is defined as an entity with a high cysteine residue content, binding to various heavy metal ions [45]. Three major species, namely *Helicobacter pylori* (*n* = 12), *Pseudomonas fluorescens* (*n* = 193), and *Pseudomonas aeruginosa* (*n* = 20), are mainly involved in metal-binding. *Pseudomonas fluorescens*, a non-pathogenic bacterium, has primarily utilized Fe^2+^ and Zn^2+^ ions in metallothionein. A specific sequence motif, **C**x**C**xx**C**, was observed for metallothionein modifications in *P. fluorescens* species [46]. Fe^2+^ was involved in iron uptake, and Zn^2+^ was involved in metallothionein and metal homeostasis [47]. *Pseudomonas aeruginosa*, a pathogenic bacterium, exhibits metal-binding to Zn^2+^, involved in zinc trafficking, and heavy metal detoxification via metallothionein, involving cysteine residues, Cys_77_, Cys_112_, and Cys_119_ [48]. The second most frequent Cys-PTM in bacteria was disulphide (Fig. 6). *S*-sulphenylation is maximally observed in bacteria (across the taxonomic world (Fig. 6), presumably because of the oxidative stress and ROS-mediated signalling that causes irreversible oxidation of the cysteine thiol group [49]. Sixty bacterial species are reported in this database those undergo *S*-sulphenylation. The bacteria-induced pathogenesis, involving disulphide, metal-binding, *S–S*-sulphenylation, and thioether, was reported earlier [50].

Almost all the viral species, reported in this database, undergo disulphide modifications (Fig. 6). Most of these viruses belong to the influenza A virus (*n* = 49 795). HA and NA proteins of influenza A virus have the maximum number of disulphide bonds (six in HA and four in NA). These disulphide bonds are involved in maintaining the structure of the proteins and facilitate viral entry (using HA) and release (using NA) [51]. Influenza virus proteins rely on the redox-dependent formation of disulphide bonds (–S–S–) for protein stability and function [52–54]. Other than disulphide, 66 viral entries were reported with lipidation (*S*-palmitoylation). All these entries belong to the hepatitis virus [55]. The core protein of the hepatitis C virus (HCV) is *S*-palmitoylated at Cys_172_ and is essential for core protein association and virus assembly in the smooth endoplasmic reticulum [56]. *S*-palmitoylation of cysteines (Cys_13_, Cys_39_, and Cys_342_) of the non-structural (NS) 5A protein and those (Cys_14_, Cys_170_, Cys_242_, and Cys_274_) of the non-structural (NS) 5B protein were involved in the hepatitis C viral RNA replication and particle assembly [57]. Cysteine *S*-palmitoylation is involved in the pathophysiological process of liver diseases and the prospective diagnostic biomarkers and therapeutic targets. Cys_261_, Cys_187_, and Cys_257_ are conserved across different hepatitis genotypes. The Cys_261_, a part of the conserved **C**xxx**C** motif in hepatitis genotypes 1a, 1b, 1c, 2a, 2b, and 3a, was present on the NS3 protease cleavage site. Similarly, Cys_257_ (part of the CxxxC motif) was conserved in different hepatitis genotypes, namely 1a, 1b, 1c, 2a, 2b, 2c, and 4a, and is mainly involved in liver pathogenesis [58].

In the case of Archaebacteria, metal-binding was most prevalent (Fig. 6). All these metal-binding PTMs belong to the metallothionein type. Metallothionein binds to heavy metal ions and is primarily involved in metal homeostasis. Disulphide was the second most prevalent PTM in Archaebacteria. For example, 40 *Natronococcus* spp. were present in this database, which have many disulphide bridges.

### Analysis of the enzyme classes across various cysteine post-translational modifications

**Figure 7:**
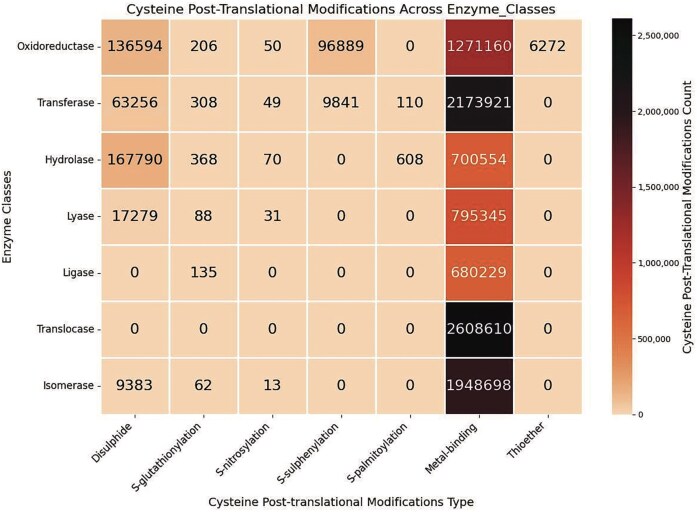
Heatmap of the cysteine post-translational modifications across enzyme classes, in this study. The figure was generated using Python (version 3.12.3) module seaborn (version 0.13.2) and matplotlib (version = 3.10.1).

Seven Cys-PTMs were classified based on enzyme classes (Fig. 7). The enzyme class (EC class) was obtained from the EC number reported by each UniProt ID; else, the EC class was left blank. Metal-binding is ubiquitous across all enzyme classes. The second most common PTM was disulphide, present in almost all the enzyme classes, except ligase and translocase. *S*-glutathionylation was also present across most PTMs, although the data size was small. Thioether was only observed in the oxidoreductase class. *S*-sulphenylation was very specific to oxidoreductase and transferase.

For the metal-binding Cys-PTM, the highest number of enzymes was from the translocase class. Conversely, the translocase enzymes reported in this database only have metal-binding Cys-PTM (Fig. 7). These translocase enzymes, mainly metalloproteins, were primarily involved in the amino acid biosynthesis biochemical pathways, etc. The metalloproteins were also observed in the isomerase enzyme class, mainly as metallochaperone. Oxidoreductase enzymes, such as dehydrogenases, oxidases, and reductases, are also metalloproteins, containing metals in their prosthetic groups. Thioether Cys-PTM was exclusively observed in oxidoreductase. This is most likely because thioether modification involves diverse ligands participating in the oxidation and reduction reactions. Oxidoreductase is essential in the thiol–disulphide exchange. Transferase enzymes are ubiquitous across the Cys-PTMs, except metal-binding; it is essential for the *S*-glutathionylation, cysteine isoprenylation, *S*-palmitoylation, *S*-farnesylation, and prenylation (different forms of lipidation) [59]. All the cysteine lipidations contain thioester bonds, hydrolysed by hydrolase enzymes [60]. Cysteine thiol is oxidized (to –SOH, *S*-sulphenylation) in the presence of oxidoreductase enzyme class, such as peroxidase. *S*-sulphenylation is also facilitated by the transferase enzyme class such as acyltransferases and malate synthase G. Oxidation of cysteine thiol to disulphide more or less involves all the classes (Fig. 7). Similarly, *S*-glutathionylation also involves the majority of the enzyme classes.

### Analysis of the cell organelles across various cysteine post-translational modifications

**Figure 8:**
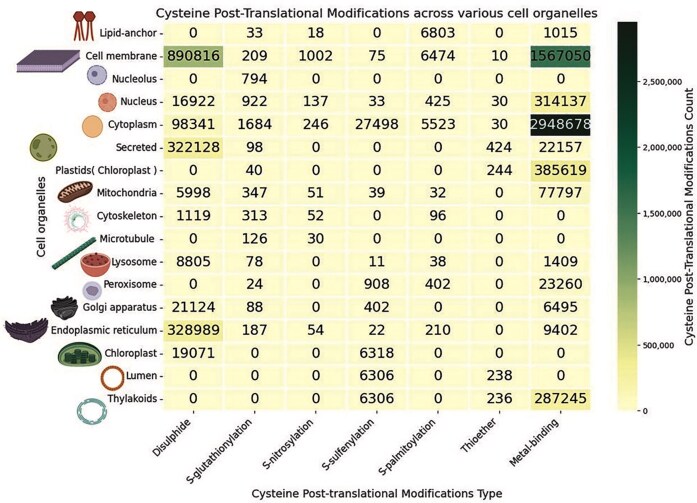
Heatmap of the cysteine post-translational modifications across various cell organelles, in this study. The figure was generated using Python (version 3.12.3) module seaborn (version = 0.13.2) and matplotlib (version = 3.10.1).

Cys-PTMs were categorized according to different cell organelles, represented by a heatmap (Fig. 8). There are other specific cell organelles from various regions and organs, with reported Cys-PTMs, such as liver and apical cells; those were not shown in the heatmap. In this study, the maximum number of Cys-PTMs was observed in the cytoplasm (*n* = 30 82 000) and cell membrane (*n* = 24 65 636). Disulphide and metal-binding were the most common Cys-PTMs in both organelles. As cytoplasm and cell membrane host many biological pathways, such as glycolysis, transportation, and secretion, the maximum number of Cys-PTMs was observed in these two organelles. Disulphide in the cytoplasm and cell membrane, reported in this database, were mostly obtained in the amyloid proteins, such as amyloid β-precursor protein, β-amyloid-like protein, amyloid-β-A4 protein, islet amyloid polypeptide (amylin), calreticulin, calnexin, and PDI proteins. These proteins were also observed in the endoplasmic reticulum and other secreted organelles (extracellular matrix, capsule, cell wall, and surface films). Cytoplasm, endoplasmic reticulum, Golgi, cell membrane, and other secreted organelles participate in transportation. The majority of the cytoplasmic and nuclear proteins are either metalloproteins or facilitate metalloprotein formation. Metal-binding PTMs were also observed in other organelles, namely the plastids and chloroplast, where metal-binding was primarily involved in the cofactor biosynthesis, plant metabolite production, plant hormone synthesis, etc. *S*-glutathionylation PTM was observed mainly in the cytoplasm and endoplasmic reticulum of hepatocytes, muscle, and cancer cells (Fig. 8). *S*-nitrosylation is primarily observed in GTPase proteins present in the cell membrane. *S*-sulphenylation is mainly observed in plant organelles as it is associated with oxidative stress-induced pathways [61]. Thioether cysteine post-translational modification is reported in very few cell organelles. Mitochondria is a significant source of energy production involving metalloproteins; thus, the metal-binding modification is most common in mitochondria. To note, mitochondrial proteins are mostly integral membrane proteins, and those are less represented in the UniProt database, so also in the current database.

### Cysteine sequence motifs representation in the CysDBase

Cysteine sequence motifs (within a 15-amino acid stretch) were explored around different cysteine PTMs within a specific species. The cysteine residue is present at the centre of the sequence motif. The motif was generated and visualized using the ‘pLogo’ web server [62]. Two inputs are required: a) foreground input: protein FASTA sequence from a specific organism, here, the 15-amino acid stretch, and b) background input: the whole proteome of the same organism. Specific cysteine PTM motifs are described below.

**Figure 9:**
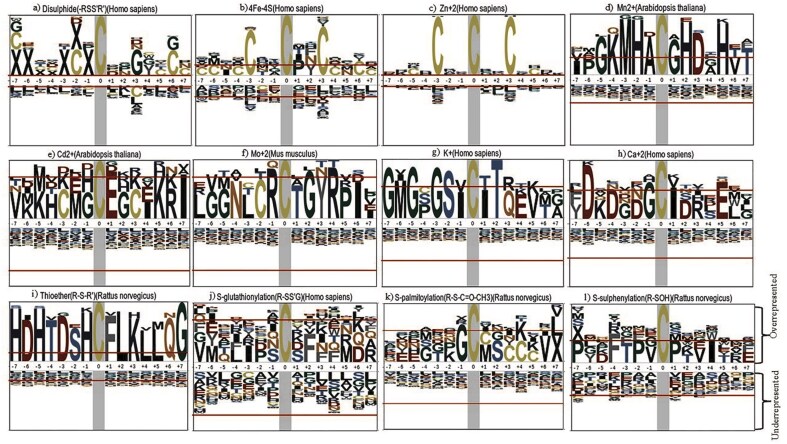
Sequence motifs around the post-translational sites of different cysteine post-translational modifications among different species: a) disulphide (*H. sapiens*), b) 4Fe–4S (*H. sapiens*), c) Zn^2+^ (*H. sapiens*), d) Mn^2+^ (*A. thaliana*), e) Cd^2+^ (*A. thaliana*), f) Mo^2+^ (*Mus musculus*), g) K^+^ (*H. sapiens*), h) Ca^2+^ (*H. sapiens*), i) thioether (*Rattus norvegicus*), j) *S*-glutathionylation (*H. sapiens*), k) *S*-palmitoylation (*H. sapiens*), and l) *S*-sulphenylation (*R. norvegicus*). The figure was generated using pLogo webserver (http://plogo.uconn.edu/). The total number of sequences considered in the (foreground [ng] and background [bg]) calculations from particular species was a) 18 277 270 204, b) 169 270 204, c) 3642 270 204, d) 10 192 010, e) 6 192 010, f) 5 265 952, g) 5 270 204, h) 7 270 204, i) 20 252 451, j) 18 270 204, k) 8 270 204, and l) 27 252451.

*Cysteine–disulphide motifs:* Cysteine disulphides (*n* = 18 277) preferred two sequence motifs, CxC and CxxGxxC (Fig. 9a). Earlier reports indicated that the disulphide bonds were present in CxxC motifs of the thioredoxin family [63]. It was reported earlier that glycine (Gly, G) at the +3 position (CxxG) is common in the disulphide sequence motif [64]. Apart from the presence of the CXXC motif in the thioredoxin family, the CXXCH motif was also reported for disulphide [65]. Note that the sequence motifs vary around PTMs from one protein family to another. In one of the earlier reports, disulphide exhibited no sequence motif [15].
*Cysteine–transition metal-binding motifs:* Cysteine metal-binding PTM was reported with several sequence motifs (Fig. 9). The metal-binding PTM motifs can be further classified into different groups; the predominant one is the transition metal ions, namely i) a 4Fe–4S cluster (*n* = 175) (Fig. 9b) and ii) Zn^2+^ ion binding (*n* = 3712) (Fig. 9c); both were embedded in the CxxCxxC motif, despite ubiquity across the enzyme classes. The Zn^2+^ ion-binding motif, CxxCxxC, belonged to coil secondary structure (Fig. S3). An earlier report showed that 30 iron-binding and 18 zinc-binding cysteines were embedded in the CxxCxxC motif [65]. A large number of other metal-binding motifs, namely CC, CxC, CxxC, and CxxxC, were also reported earlier [65]
*New preliminary observations on cysteine metal-binding motifs:* A few other motifs were identified, i) around Mn^2+^ (*n* = 10): MHxCxHD motif (Fig. 9d), ii) Cd^2+^ (*n* = 6) (Fig. 9e), iii) Mo^2+^ (*n* = 5) (Fig. 9f), iii) K^+^ (*n* = 5): GSxCxT motif (Fig. 9g), and iv) Ca^2+^ (*n* = 7): CxxCExxxK (Fig. 9h). These motifs cannot be validated as they have not been reported earlier. The data sizes of these motifs are too small for statistical inference; hence, we label these motifs as preliminary observational inferences. These preliminary observations are subject to further experimental validation. Mn^2+^ ion, being a transition metal ion, like Fe^2+^ and Zn^2+^, showed a specific motif signature where the central cysteine is flanked by histidine residues on both sides, possibly indicating coordination to a metal ion. In contrast to transition metal ions, alkali (K^+^) and alkaline earth (Ca^2+^) metal ions do not have specific coordination chemistry but participate in non-specific interactions involving negatively charged or polar groups.
*New cysteine–thioether motif:* The motif around thioether PTMs is reported for the first time (Fig. 9i) and cannot be validated. Thioether cysteine modification reported here (*n* = 20) mostly belonged to the oxidoreductase enzyme class (cysteine dioxygenase family) that showed a specific sequence motif, HCFLK.
*Cysteine PTMs without defined motifs:* There are some Cys-PTMs reported without any sequence motif signatures; however, they are enriched with certain types of amino acids in the vicinity. For example, *S*-glutathionylation (Fig. 9j), *S*-palmitoylation (Fig. 9k), and *S*-sulphenylation (Fig. 9l) — all these cysteine residues belonged to coil secondary structures (Fig. S4). *S*-glutathionylation and *S*-palmitoylation PTMs are rich in phenylalanine in the vicinity, supported by the literature, where hydrophobic amino acids were enriched in *S*-glutathionylation cysteine regions [66]. Another research work reported CC and CxC motifs for *S*-palmitoylation and acidic amino acid-enriched motifs for *S*-glutathionylation [15]. The current study showed proline-enriched motifs for *S*-sulphenylation; the proline residue was evolutionarily conserved in many species, including *H. sapiens* and *A. thaliana* [67]. Literature reports showed that sequence motifs around *S*-sulphenylation, *S*-sulfhydration, and *S*-sulfinylation were enriched in basic amino acids, such as lysine [15]. Other Cys-PTMs, with less featured sequence motifs, were reported in the supplementary material (Fig. S4a–f). For example, Ni^2+^, Fe^2+^, and Cu^2+^ ions, despite being transition metal ions, have shown almost no sequence conservation patterns in *Escherichia coli* species (Fig. S4a–c). To note, metallothioneins, rich in cysteine, showed a specific signature of repeating cysteines (Fig. S4g).

### Analysis of the protein microenvironments (MENV) around the cysteine post-translational modifications (PTMs)

**Figure 10:**
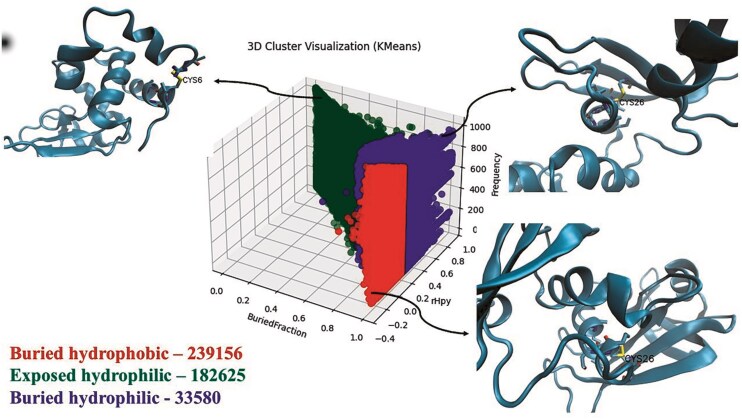
Three clusters of protein microenvironments (defined by buried fraction [X-axis] and rHpy [Y-axis]) around cysteines reported in this database. Frequencies of each cluster are shown, following the cluster names. A representative subset (10% of the total data points) was used for clustering. Three-dimensional representations of the microenvironments are shown in the insets: buried hydrophobic (PDB ID: 11BA), buried hydrophilic (PDB ID: 11BG), and exposed hydrophilic (PDB ID: 132L). The figure was generated using i) matplotlib, ii) VMD, and iii) Microsoft ‘PowerPoint’ 365 suite.

The protein microenvironments (MENV) around the Cys-PTMs were computed and clustered into three groups using the K-means clustering method [28]. The details of the clusters were shown (Table S4). The largest was buried hydrophobic, and the smallest was exposed hydrophilic (Fig. 10).

**Figure 11:**
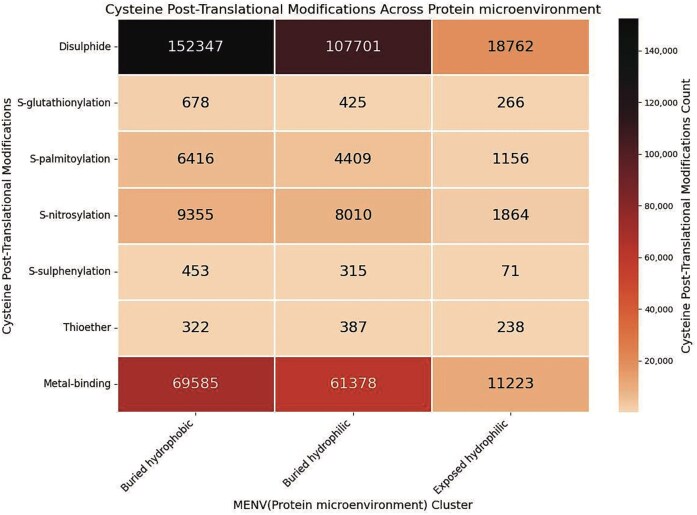
Heatmap of the cysteine post-translational modifications across the three microenvironment clusters, in this study. The figure was generated using Python (version 3.12.3) module seaborn (version 0.13.2) and matplotlib (version = 3.10.1).

Although the exposed hydrophilic group was the smallest in size, the number of cysteines in this group was significantly large (Fig. 10). Protein microenvironment is an essential modulator of protein structure and functions, as demonstrated earlier. Cysteine residues are primarily embedded in the buried hydrophobic microenvironment, showing the least preference towards the exposed hydrophilic protein microenvironment [11,65]. An earlier study reported that the amino acids embedded in a mismatched microenvironment (e.g. cysteine in the exposed hydrophilic microenvironment) mostly demonstrate structural or functional consequences [27]. Cys-PTMs evolved as required for structural stabilization or protein functions. These structural or functional requirements most likely exposed the cysteine residues to the hydrophilic microenvironment. For example, Cys_149_ from GapC1 protein in *A. thaliana* is exposed to the solvent (hydrophilic microenvironment) and prone to irreversible oxidation by H_2_O_2_, inhibiting enzyme activity. In the presence of the glutathione molecule, Cys_149_ undergoes *S*-glutathionylation, thus preventing further oxidation of the thiol group [38]. Most of the cysteine residues in the exposed hydrophilic protein microenvironment exhibited disulphide or metal-binding PTMs (Fig. 11). Most of the disulphides in the exposed hydrophilic microenvironments belonged to secreted and cytoplasmic proteins from certain viruses, namely hepatitis virus (*n* = 1987), SARS-CoV-2 virus (*n* = 22 386), and HIV (*n* = 874). These disulphides undergo thiol–disulphide exchange, glutathionylation, further oxidations, etc. Metal-binding cysteines were mainly part of iron–sulphur clusters (*n* = 3357) from electron transport chains, where cysteine residues were exposed. Some of the cysteines from polyproteins of the viruses, such as HCV (*n* = 3055) and HIV (*n* = 1350), were exposed to the protein surface.

### Contemporary cysteine databases

The database developed in this work (CysDBase) was compared with 16 contemporary databases and web servers; some of those were not in their working conditions (Table S5). No databases were available to compare with thioether.

*Single cysteine prediction databases:* Cysteine disulphide, disulphide bridges, or disulphide patterns were reported in the following databases, namely DBDB [68], DSDBASE2.0 [69], and Cys.sqlite [19]. The function of the *S*-glutathionylated proteins and their structural analysis were curated in the dbGSH database [12]. The structure, function, and motif analysis for *S*-nitrosylation were curated in the dbSNO2.0 database [13]. The Metal S^3^ database stores information on Fe–S clusters and predicts metal ion sites from sequences [70].
*Multiple cysteine PTM databases:* Currently available multiple Cys-PTM databases are RedoxDB, iCysMod, CysModDB, and qPTMPants. RedoxDB contained information on different cysteine-oxidized products: disulphide, *S*-nitrosylation, *S*-glutathionylation, *S*-sulphenylation, *S*-sulphonation, and others [71]. iCysMod has curated information from 48 eukaryotes on disulphide, *S*-glutathionylation, *S*-nitrosylation, *S*-sulfhydration, *S*-sulphenylation, *S*-sulfinylation, and *S*-sulfonylation [15]. CysModDB contained 12 different Cys-PTMs, namely, *S*-nitrosylation, *S*-sulphenylation, *S*-sulfinylation, *S*-sulfonylation, *S*-glutathionylation, disulphide, *S*-persulfidation, *S*-palmitoylation, *S*-prenylation, *S*-carbonylation, *S*-itaconation, and *S*-succination. *S*-itaconation is a covalent modification of cysteine where metabolite itaconate was added via the Michael addition. *S*-succination, *S*-(2-succino) cysteine, is reported here from 12 different species [16]. qPTMplants, an integrative database of quantitative Cys-PTMs, contained Cys-PTMs, namely *S*-persulfidation, *S*-nitrosylation, and *S*-sulphenylation, from 43 different plant species [17].

Another set of databases includes multiple PTMs for a large number of amino acids (including cysteine), namely dbPTM, dbPTM3.0, dbPTM2022, dbPTM2025, etc. dbPTM contained Cys-PTMs, namely, *S*-palmitoylation, disulphide, *S*-glutathionylation, thioether, *S*-sulfhydration, and *S*-nitrosylation [72]. That was later updated to dbPTM3.0 with additional information on substrate specificity and functional association of the Cys-PTMs [18]. The database was further updated to dbPTM2022 with additional information on regulatory networks of the Cys-PTMs [73]. The latest upgradation of the database, dbPTM2025, contained additional information on the proteomics data of cancer research for the same Cys-PTMs [74].

*Other cysteine databases:* Other cysteine databases contained various additional cysteine information, such as the Cysteine Motif Database (CMD) [14], Cysteine annotation database, Cysteinome [75], experimental chemo proteomics databases, CysDB [20], and TopCysteineDB [76].

**Web server and standalone programme:**

**Figure 12:**
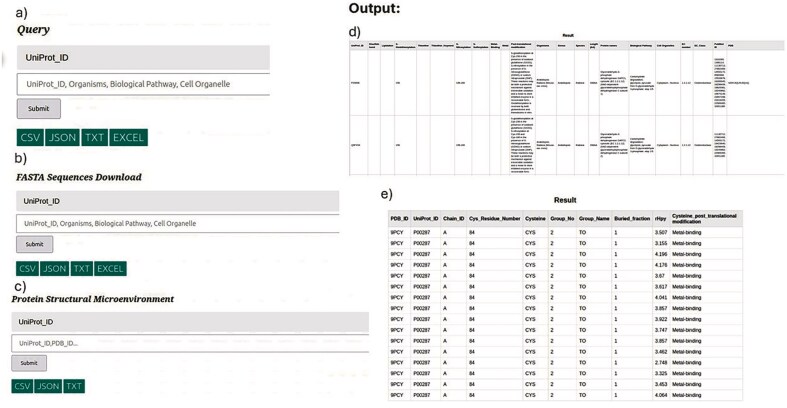
(Left panel)Query: CysDBase Online Webserver page Input: a) General Query Section, b) FASTA Sequences Download, and c) protein structural microenvironment. (Right panel) Outputs: d) Biochemical information (tabular form) and e) protein microenvironment.

***Query input***:

The input for the CysDBase database webserver consists of three sections:

*The first section* is a general query section where the input can be UniProt_ID, organisms, biological pathway, or cell organelle. Any of these queries will result in details of seven Cys-PTMs (Fig. 12a).
*The second section* instructs to download the FASTA sequences using the same queries, as above (Fig. 12b). The purpose of delineating first and second sections is two-fold: i) the internal database file becomes too long when FASTA information is included in the general query section, hence, difficult for I/O operation, and ii) delineating sections, one and two, simplifies the search for different usages, e.g. bulk sequence download versus biological information.
*The third section:* Protein structure and microenvironment information can be downloaded separately from the CysDBase using PDB or UniProt IDs queries. The protein microenvironment will be reported for UniProt IDs, provided the corresponding PDB IDs are available (Fig. 12c).

Three separate CSV files are maintained for three different sections in the internal database.

***Query output***

Three query outputs corresponding to the three inputs mentioned above can be produced. The results from the input types one and three can be downloaded in tabular form (CSV, JSON, and TXT) and viewed in a separate HTML page (Fig. 12d and e). The FASTA sequences can only be downloaded in CSV format.

**Database download policy:**

The CysDBase can be downloaded upon request via email and prior approval.

**Documentation:**

Technical documentation is available on the web server, including the terms and conditions, copyright, disclaimer notice, procedure to register to the CysDBase, hardware/software requirements, etc.
Programmatic documentation is available in the GitHub repository. The documents explain the Python code used to extract queries and develop the database. The GitHub repository also comprises programmatic access to the CysDBase database web server. The query search can be accessed from GitHub using the Python program or Microsoft Excel functions and downloaded as a CSV file. Input queries and corresponding outputs are identical to those in the web server.

**Web-applications security, maintenance, sustainability, standardization, and community support:**

Web applications were secured using a Secure Sockets Layer (SSL) certificate that provides a trusted Certificate Authority (CA), which was derived from Let’s Encrypt (https://letsencrypt.org/) by using Certbot (https://certbot.eff.org/). The web application was converted from Hypertext Transfer Protocol (HTTP) to Hypertext Transfer Protocol Secure (HTTPS), reducing traffic control. SSL ensures secure communication between the web application and the client. There is also an HTTPS/TLS (Transport Layer Security) encryption, which encrypts communication by converting it into code between the web application and the client to provide a private and secure connection. The web application was hosted in the Ubuntu operating system (Ubuntu 22.04.5) for security, where it has a universal firewall (UFW). The universal firewall was activated when hosting the web application, creating an IPv4 and IPv6 host-based firewall. IPv4 and IPv6 internet protocols are the most secure and provide confidentiality and data integrity. Web applications were maintained by restarting when the FLASK files were updated. A pip (20.0.2) package manager and virtual environments managed all the FLASK application packages to ensure consistency across the deployments. WSGI servers are monitored and are restarted when any new version is deployed. NGINX and WSGI servers are configured to generate logs for troubleshooting and monitoring. Sustainability of the web application was maintained by compressing the files to reduce the size and improve the loading times. The web application was standardized by setting up a consistent process, creating a WSGI server like Gunicorn, which creates a virtual environment, configuring NGINX as a reverse proxy, and securing the application with HTTPS. The community supported the web application, where proper documentation was made, clear code organization was maintained, and active participation from online communities such as Stack Overflow, Reddit, and GitHub was open. The questions from the community are addressed, and advice from other developers is followed. The web application is open to feedback, suggestions, and collaboration from different developers to improve the web application. If there are any new features and bugfixes, they are announced to the community.

## Supplementary Material

baag021_Supplemental_Files

## Data Availability

The data and software are available in the following GitHub link: https://github.com/devhimd19/CysDBase.
