# Supplementary material for: CysDBase: a comprehensive database of cysteine post-translational modifications across protein sequence, structure, microenvironment, class, cellular localization, biological pathway, and taxonomy
Source: Database (Oxford). 2026 May 12;2026:baag021. doi: 10.1093/database/baag021 (PMC13161762; doi:10.1093/database/baag021)
Supplement: baag021_Supplemental_Files [file baag021_supplemental_files.zip › Table S1.docx]

Table S1: Keywords for thioether curation from the UniProt database

| S-methylcysteine |
| --- |
| Lanthionine (Cys-Ser) |
| Beta-methyllanthionine (Thr-Cys) |
| S-(2-aminovinyl)-3-methyl-D-Cysteine (Cys-Cys) |
| 5-amino-piperidine-2,5-dicarboxylic acid (Ser-Cys) |
| (4S)-Thiazoline-4-carboxylic acid |
| Thiazole-4-carboxylic acid (Asn-Cys) |
| Thiazole-4-carboxylic acid (Phe-Cys) |
| Thiazole-4-carboxylic acid (Ile-Cys) |
| Thiazole-4-carboxylic acid (Cys-Cys) |
| Thiazole-4-carboxylic acid (Ser-Cys) |
| Thiazole-4-carboxylic acid (Thr-Cys) |
| Pyridine-2,5-dicarboxylic acid (Ser-Cys) |
| 2,3-didehydroalanine (Cys) |
| 2-(S-cysteinyl)-Histidine (Cys-His) |
| 3-(S-cysteinyl)-Tyrosine (Cys-Tyr) |
| 6-(S-cysteinyl)-8alpha-(prohistidyl)-FAD (His-Cys) |
| 4-cysteinyl-glutamic acid (Cys-Glu) |
| 4-cysteinyl-Aspartic acid (Cys-Asp) |
| 3-cysteinyl-aspartic acid (Cys-Asp) |
| 4^\|^-cysteinyl-tryptophylquinone (Cys-Trp) |
| Cyclopeptide (Cys-Arg) |
| S-(2,3-dicarboxypropyl) cysteine |
| S-8alpha-FAD cysteine |
| N, N-(cysteine-1,S-diyl)phenylalanine (Cys-Phe) |
| 2'-(S-cysteinyl)-histidine (Cys-His) |
| S-cysteinyl 3-(oxidosulfanyl)alanine (Cys-Cys) |
