# Supplementary material for: CysDBase: a comprehensive database of cysteine post-translational modifications across protein sequence, structure, microenvironment, class, cellular localization, biological pathway, and taxonomy
Source: Database (Oxford). 2026 May 12;2026:baag021. doi: 10.1093/database/baag021 (PMC13161762; doi:10.1093/database/baag021)
Supplement: baag021_Supplemental_Files [file baag021_supplemental_files.zip › Table S2.docx]

Table S2: Different types of existence of protein evidence curated from UniProt database corresponding to various Cys-PTMs. Percentage of each type of protein evidence is given in parenthesis

| **Cys-PTM** | **Protein_level** | **Transcript level** | **Homology** | **Predicted** | **Uncertain** |
| --- | --- | --- | --- | --- | --- |
| Thioether(n=6070) | 120 (0.019) | 195 (0.032) | 4497 (0.740) | 2 (0.0003) | 1252 (0.206) |
| S-glutathionylation(n=1494) | 1457 (0.975) | 19 (0.012) | 16 (0.010) | 1 (0.0006) | 1 (6.69E-04) |
| S-palmitoylation(n=7158) | 683 (0.095) | 383 (0.053) | 4626 (0.646) | 1322 (0.184) | 142 (0.019) |
| S-nitrosylation(n=911) | 349 (0.383) | 132 (0.144) | 390 (0.428) | 31 (0.034) | 5 (0.005) |
| Disulphide(n=1492807) | 24945 (0.016) | 40202 (0.026) | 982563 (0.658) | 443528 (0.297) | 1569 (0.001) |
| S-sulphenylation(n=143802) | 279 (0.001) | 822 (0.005) | 66862 (0.464) | 4224 (0.029) | - |

| Metal_binding (Total n= 1863288) |  |  |  |  |  |
| --- | --- | --- | --- | --- | --- |
| Ca(n=21408) | 247 (0.011) | 278 (0.012) | 2732 (0.127) | 17565 (0.820) | 578 (0.026) |
| Co(n=7) | 4 (0.571) | - | 3 (0.428) | - | - |
| Cd(n=38) | 22 (0.578) | 1 (0.026) | 14 (0.368) | - | - |
| 8Fe-7S(n=60) | 12 (0.2) | - | 48 (0.8) | - | - |
| 7Fe-Mo(n=31) | 3 (0.096) | - | 28 (0.903) | - | - |
| Hg(n=48) | 10 (0.208) | 1 (0.020) | 28 (0.583) | 8 (0.167) | - |
| Metal-thiolate cluster(n=179) | 55 (0.307) | 39 (0.217) | 84 (0.469) | - | 1 (0.005) |
| 3Fe-4S(n=13752) | 115 (0.008) | 42 (0.003) | 7741 (0.562) | 385 (0.027) | 5466 (0.397) |
| Cu(n=62812) | 255 (0.004) | 377 (0.006) | 27154 (0.432) | 4085 (0.065) | 2 (0.00003) |
| Ni-4Fe-4S(n=3563) | 15 (0.004) | - | 473 (0.132) | - | 2126 (0.596) |
| Ni-4Fe-5S(n=9) | 2 (0.222) | - | 7 (0.777) | - | - |
| Ni-Fe-S(n=503) | 3 (0.005) | - | 178 (0.353) | - | 322 (0.640) |
| Ni(n=31913) | 57 (0.001) | 3 (0.00009) | 10747 (0.336) | 3010 (0.094) | - |
| K(n=49839) | 121 (0.002) | 146 (0.002) | 28107 (0.563) | 149 (0.002) | 1 (0.00002) |
| 2Fe-2S(n=92227) | 654 (0.007) | 560 (0.006) | 72313 (0.784) | 4996 (0.054) | 13704 (0.148) |
| 4Fe-4S(n=811796) | 1197 (0.001) | 1128 (0.001) | 436808 (0.538) | 11675 (0.014) | 360988 (0.444) |
| Zn(n=775103) | 4629 (0.005) | 4858 (0.006) | 682586 (0.880) | 83027 (0.107) | 3 (0.000003) |
