# Supplementary material for: CysDBase: a comprehensive database of cysteine post-translational modifications across protein sequence, structure, microenvironment, class, cellular localization, biological pathway, and taxonomy
Source: Database (Oxford). 2026 May 12;2026:baag021. doi: 10.1093/database/baag021 (PMC13161762; doi:10.1093/database/baag021)
Supplement: baag021_Supplemental_Files [file baag021_supplemental_files.zip › Table S3.docx]

Table S3: Crosstalk of pairwise and multiple cysteine post-translational modifications with the information of Protein name, Cell organelles and Organism.

| Type of multiple cysteine post-translational modification. | UniProt_ID | Cys_Residue | Protein Name | Cell Organelles | Organism |
| --- | --- | --- | --- | --- | --- |
| 2Fe-2S Metal-binding, S-glutathionylation | Q84Y95 | 97 | Monothiol glutaredoxin | Plastid,chloroplast | Arabidopsis thaliana |
| Disulphide, S-nitrosylation | P27695 | 93 | DNA repair nuclease/redox regulator APEX1 | Nucleus,Endoplasmic reticulum,Cytoplasm | Homo sapiens |
| Cu-Metal-binding, Disulphide | A0A9W8DSZ4 | 1763 | Pentafunctional AROM polypeptide | Cytoplasm | Mycoemilia scoparia |
| Fe-Metal-binding, Disulphide | Q8ZPH0 | 79 | Uptake hydrogenase large subunit | Cell envelope | Salmonella typhimurium |
| Fe-Metal-binding,Ni-Metal-binding, Disulphide,S-sulphenylation | Q72AS3 | 78 | Periplasmic [NiFeSe] hydrogenase, large subunit, selenocysteine-containing | Cell envelope | Nitratidesulfovibrio vulgaris |
| Na-Metal-binding, Disulphide | O35305 | 134 | Tumor necrosis factor receptor superfamily member 11A | Cell membrane | Mus musculus |
| Thioether, Ca-Metal-binding | A0A087Y0M9 | 88 | Extracellular sulfatase | Endoplasmic reticulum,Golgi apparatus,Golgi stack,Cell surface | Poecilia formosa |
| Disulphide, Ca-Metal-binding | A0MSJ1 | 1511 | Collagen alpha-1(XXVII) chain B | Secreted, extracellular space, extracellular matrix | Danio rerio |
| Mn-Metal-binding, Thioether | Q1M964 | 57 | Multifunctional alkaline phosphatase superfamily protein pRL90232 | Cytoplasm | Rhizobium johnstonii |
| Disulphide,Mn-Metal-binding | Q9I000 | 69 | Phospho-2-dehydro-3-deoxyheptonate aldolase | - | Pseudomonas aeruginosa |
| S-glutathionylation, Thioether | P05813 | 82;117 | Beta-crystallin A3 | Cytoplasm, Nucleus | Homo sapiens |
| Disulphide,S-glutathionylation,S-nitrosylation,S-sulphenylation | P0ACQ4 | 199 | DNA-binding transcriptional dual regulator OxyR | Cytosol, Protein-DNA complex | Escherichia coli |
| S-glutathionylation,S-nitrosylation | P25858 | 156 | Glyceraldehyde-3-phosphate dehydrogenase GAPC1 | Cytoplasm, Nucleus | Arabidopsis thaliana |
| S-palmitoylation, S-glutathionylation | I3LMB3 | 62 | Phospholemman | Cell membrane | Sus scrofa |
| Disulphide,S-glutathionylation | O00299 | 24 | Chloride intracellular channel protein 1 | Nucleus,Endoplasmic reticulum,Cytoplasm,Cell membrane | Homo sapiens |
| S-palmitoylation, S-nitrosylation | P19838 | 61 | Nuclear factor NF-kappa-B p105 subunit | Cytoplasm, Nucleus | Homo sapiens |
| Disulphide,S-palmitoylation,S-glutathionylation | P30044 | 100 | Peroxiredoxin-5, mitochondrial | Mitochondrion,Cytoplasm,Peroxisome | Homo sapiens |
| S-glutathionylation,Thioether, S-nitrosylation, S-sulphenylation | P18031 | 215 | Tyrosine-protein phosphatase non-receptor type 1 | Endoplasmic reticulum membrane | Homo sapiens |
| S-nitrosylation, Zn-Metal-binding | O08557 | 274 | N(G),N(G)-dimethylarginine dimethylaminohydrolase 1 |  | Rattus norvegicus |
| Disulphide,S-nitrosylation | A0A0G2QC33 | 292 | Cysteine protease ATG4B | Cytoplasm,Autophagosome,Endoplasmic reticulum,Mitochondrion | Rattus norvegicus |
| Disulphide,S-sulphenylation | A0A0K3AUJ9 | 55 | Peroxiredoxin prdx-2 | Cytoplasm | Caenorhabditis elegans |
| Thioether,S-sulphenylation | P18031 | 215 | Tyrosine-protein phosphatase non-receptor type 1 | Endoplasmic reticulum membrane,Peripheral membrane protein,Cytoplasmic side | Homo sapiens |
| S-sulphenylation, Fe-Metal-binding | P13448 | 115 | Nitrile hydratase subunit alpha |  | Rhodococcus erythropolis |
