# Supplementary material for: CysDBase: a comprehensive database of cysteine post-translational modifications across protein sequence, structure, microenvironment, class, cellular localization, biological pathway, and taxonomy
Source: Database (Oxford). 2026 May 12;2026:baag021. doi: 10.1093/database/baag021 (PMC13161762; doi:10.1093/database/baag021)
Supplement: baag021_Supplemental_Files [file baag021_supplemental_files.zip › Table S4.docx]

Table S4: Statistics (average value) of cysteine microenvironment clusters. Standard deviation (**σ)** is given in parenthesis.

| Cluster type | Average Buried Fraction (**σ)** | Average rHpy (**σ)** | Average distance centroid (Å) | Number of cysteines in each cluster. |
| --- | --- | --- | --- | --- |
| Buried hydrophobic | 0.98(0.02) | 0.02(0.09) | 0.09 | 239156 |
| Buried hydrophilic | 0.90(0.08) | 0.29(0.10) | 0.12 | 182625 |
| Exposed hydrophilic | 0.51(0.16) | 0.55(0.14) | 0.19 | 33580 |
