# Supplementary material for: CysDBase: a comprehensive database of cysteine post-translational modifications across protein sequence, structure, microenvironment, class, cellular localization, biological pathway, and taxonomy
Source: Database (Oxford). 2026 May 12;2026:baag021. doi: 10.1093/database/baag021 (PMC13161762; doi:10.1093/database/baag021)
Supplement: baag021_Supplemental_Files [file baag021_supplemental_files.zip › Table S5.docx]

Table S5: Cysteine contemporary databases containing the cysteine post-translational modifications:

| **Database**  Features | Number of proteins | Number of cysteine Residues |
| --- | --- | --- |
| Disulphide bonding patterns | 10,568 | 41,846 |
| **dbPTM**  S-nitrosylation,S-palmitoylation | 35,947 | 5,272 |
| **DBDB**  Disulphide bridges | 356 | 2,042 |
| **dbPTM 3.0**  S-palmitoylation,Prenylation,S-nitrosylation,S-glutathionylation | 35,835 | 50,909 |
| **Cysteine Motifs Database (CMD)**  Flanking motifs with their secondary structure and propensity values | 1,74,597 | 8,78,000 cysteine motifs |
| **RedoxDB**  Disulphide,S-nitrosylation,S-glutathionylation,Sulphination,Unknown modifications with the information of Gene name, Description, Organism and Length | 2,315 | 2,308 |
| **dbGSH**  Database of S-glutathionylation with structural, functional motifs, solvent accessibility, secondary, tertiary structures, protein domains and gene ontology | 1,314 | 2,257 |
| **dbSNO2.0**  Database of S-nitrosylation for structural, functional, disease associated and regulatory network. | 2,277 | 4,165 |
| **Cysteinome**  Display, search and analysis of the structure, function and related annotation for proteins with targetable cysteine as well as their covalent modulators. | 462 | 1,217 |
| **Cys.sqlite**  Cysteine disulphide bonds | 95,000 | 500K Cysteines as structural conformers, and 265K disulfide bond conformations. |
| **iCysMod**  Eukaryotes for S-glutathionylation, Oxidation, S-nitrosylation, Disulphide, S-sulfhydration, S-nitrosylation, S-sulfinylation and S-palmitoylation | 31,843 | 85,747 |
| **CysModDB**  S-nitrosylation, S-sulfenylation,S-sulfinylation,S-sulfonylation, S-glutathionylation,Disulphide, S-persulfidation, S-palmitoyolation | 21,654 | 70,536 |
| **DSDBASE2.0**  Disulphide bonds | 1,53,944 | 2,16,096 – Native disulphides and 2,01,53,850 – Modelled disulphides |
| **dbPTM2022**  Regulatory networks and functional association of post-translational modifications. | 15,289 |  |
| **qPTMplants**  Quantative cysteine post-translational modifications in plants | 3,425 | 5,314 |
| **CysDB**  A human cysteine database based on experimental quantitative chemoproteomics | 11,621 | 62,888 |
| **dbPTM 2025**  substrate site specificity, functional association with S-glutathionylation, S-diacylglycerol and S-nitrosylation | 2,950 | 7,204 |
| **TopCysteineDB**  PDB structural information with chemoproteomics data obtained through experiments. | 1,92,716 | 2,64,234 |
