# Supplementary figures and images for: CysDBase: a comprehensive database of cysteine post-translational modifications across protein sequence, structure, microenvironment, class, cellular localization, biological pathway, and taxonomy
Source: Database (Oxford). 2026 May 12;2026:baag021. doi: 10.1093/database/baag021 (PMC13161762; doi:10.1093/database/baag021)

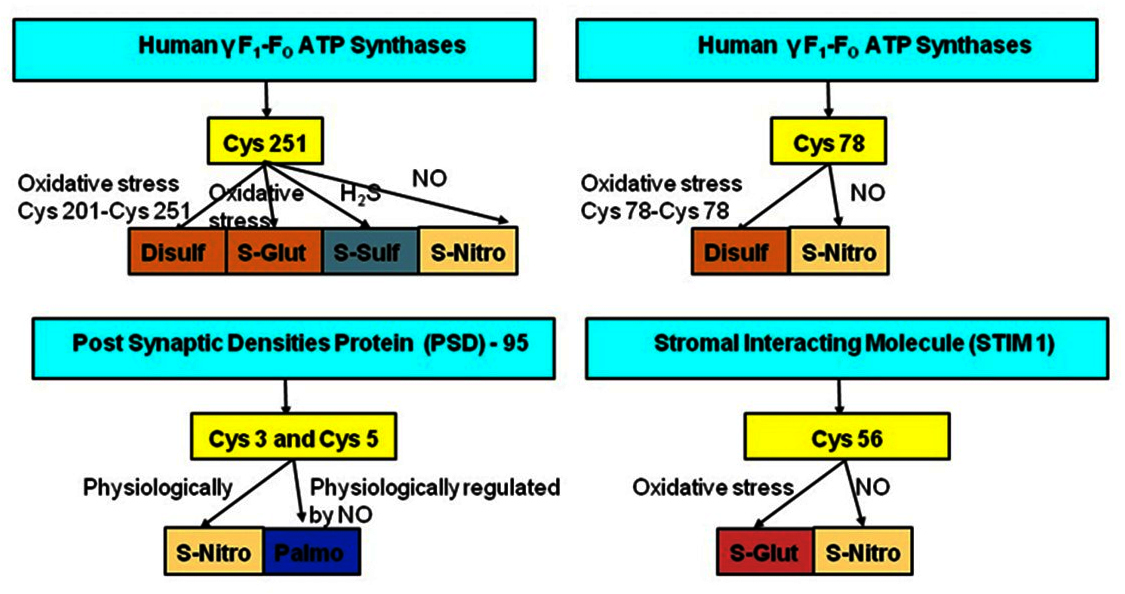

Supplement: baag021_Supplemental_Files [file baag021_supplemental_files.zip › Figure_S1.jpg]

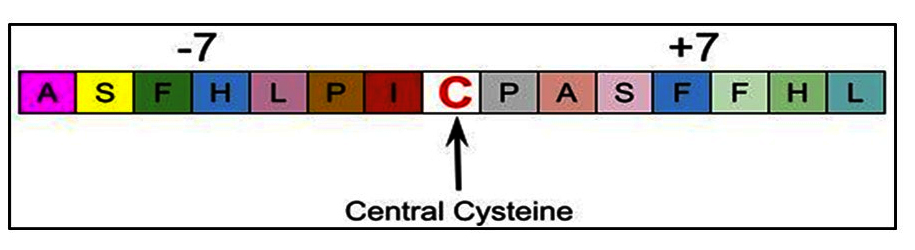

Supplement: baag021_Supplemental_Files [file baag021_supplemental_files.zip › Figure_S2.jpg]

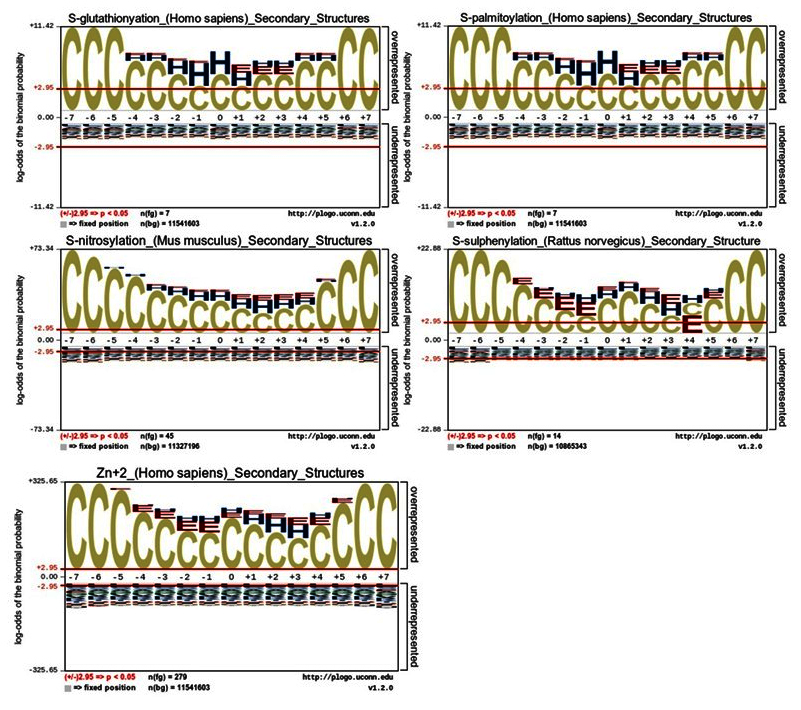

Supplement: baag021_Supplemental_Files [file baag021_supplemental_files.zip › Figure_S3.jpg]

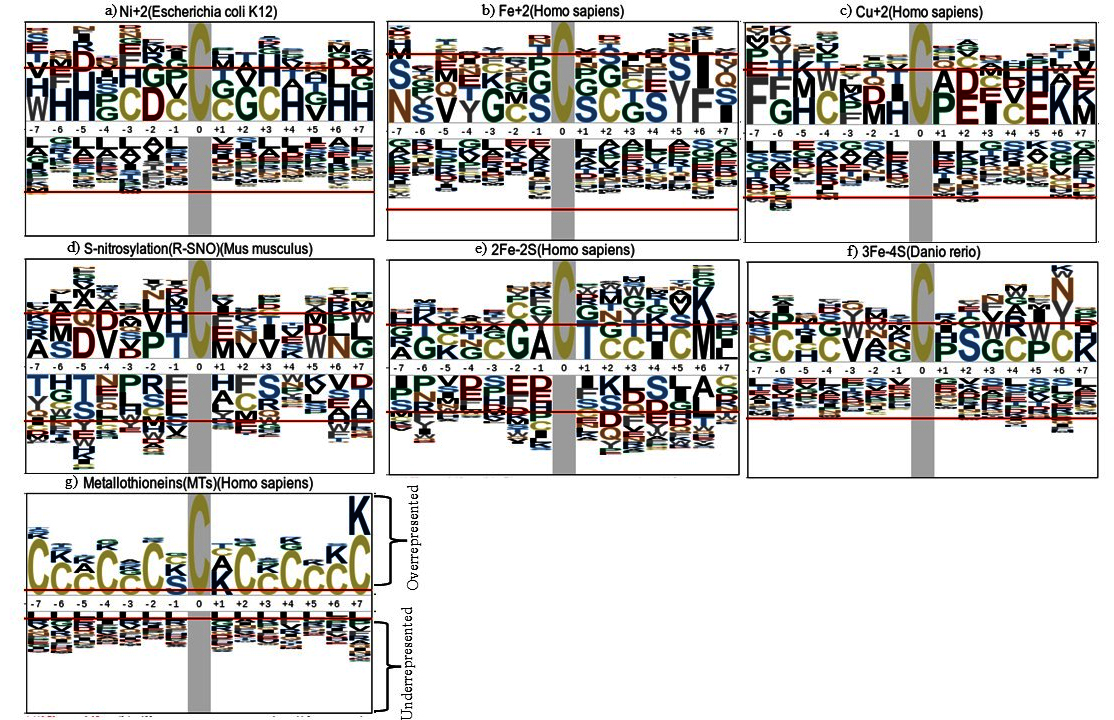

Supplement: baag021_Supplemental_Files [file baag021_supplemental_files.zip › Figure_S4.jpg]
